# Multiple lymphomatous polyposis of the gastrointestinal tract

**DOI:** 10.1590/S1516-31802004000300011

**Published:** 2004-05-06

**Authors:** Maria Isete Fares Franco, Jaques Waisberg, Leonardo Seligra Lopes

**Keywords:** Mantle-cell lymphoma, Non-Hodgkin lymphoma, Lymphoma, Linfoma de célula do manto, Linfoma não Hodgkin, Linfoma

## Abstract

**CONTEXT:**

Gastrointestinal multiple lymphomatous polyposis is a rare type of malignant lymphoma that has aggressive biological behavior, early systemic dissemination and poor prognosis. It is considered to be a manifestation of non-Hodgkin lymphoma and represents the gastrointestinal counterpart of mantle cell nodal lymphoma.

**OBJECTIVE:**

A case of gastrointestinal multiple lymphomatous polyposis is presented and the anatomopathological, clinical, diagnostic and treatment aspects of this unusual neoplasia are discussed.

**CASE REPORT:**

The patient was a 59-year-old white male with a complaint of asthenia, night sweating, alteration in intestinal habit and weight loss over the preceding two months. The physical examination showed pallid mucosa and a palpable mass in the epigastrium and mesogastrium. Endoscopy of the upper digestive tract showed the presence of gastric and duodenal polyps. An opaque enema showed multiple polypoid lesions, especially in the cecum. A rectal biopsy revealed infiltration of the mucosa and submucosa by diffuse lymphoma consisting of small cleaved cells. Immunohistochemical study showed lymphocytes that expressed the antibody CD20 (L-26) and light-chain kappa (k) immunoglobulin, but not light-chain lambda (l) immunoglobulin. The patient presented a condition of acute intestinal obstruction with the presence of a mesenteric mass formed by agglutinated lymph nodes that surrounded the proximal ileum, thereby obstructing its lumen. He was submitted to a segmental enterectomy and gastrotomy with excisional biopsies of the gastric polypoid lesions. After two cycles of chemotherapy there was a worsening of the general state, with an increase in the dimensions of the abdominal masses and sepsis, accompanied by progressive respiratory insufficiency, leading to death.

## RESUMO

**CONTEXTO:**

A polipose linfomatosa múltipla gastrintestinal é tipo raro de linfoma maligno de comportamento biológico agressivo, com disseminação sistêmica precoce e prognóstico sombrio. A polipose linfomatosa múltipla gastrintestinal é considerada manifestação do linfoma não-Hodgkin e representa a contraparte gastrintestinal do linfoma nodal das células do manto.

**OBJETIVO:**

O presente estudo descreve caso de polipose linfomatosa múltipla gastrintestinal e discute os aspectos anatomopatológicos, clínicos, diagnósticos e terapêuticos desta inusitada neoplasia.

**RELATO DE CASO:**

Homem de 59 anos, bran-co, apresentou-se ao nosso serviço com queixa de astenia, suores noturnos, alteração do hábito intestinal e emagrecimento há dois meses. O exame físico mostrou mucosa descorada e massa palpável no epigástrio e mesogástrio. A endoscopia digestiva alta revelou pólipos gástricos e duodenais. O enema opaco mostrou lesões polipóides múltiplas, principalmente no ceco. A biópsia retal revelou infiltração da mucosa e submucosa por linfoma difuso de células pequenas clivadas. O estudo imuno-histoquímico mostrou linfócitos que expressavam o anticorpo CD20 (L26) e imunoglobulina de cadeia leve kappa (k), porém não expressava imunoglobulina de cadeia leve lambda (λ). O doente apresentou abdome agudo obstrutivo devido a massa mesentérica formada por linfonodos aglutinados ao redor do íleo proximal e obstruindo seu lúmen. O enfermo foi submetido a enterectomia segmentar e gastrotomia com biópsias excisionais das lesões polipóides gástricas. Após dois ciclos de quimioterapia, houve piora do estado geral, com aumento das dimensões das massas abdominais e sepse, acompanhada por insuficiência respiratória progressiva e óbito.

## INTRODUCTION

Diffuse polypoid lymphoma that selectively involves the lymphoid tissue of the gastrointestinal tract is named gastrointestinal multiple lymphomatous polyposis.^1^ It is considered to be a rare manifestation of non-Hodgkin lymphoma and represents the gastrointestinal counterpart of nodal mantle cell lymphoma.^2^ The pattern displayed consists of centrocytic lymphoma with a tendency towards early systemic dissemination and an aggressive clinical course.^2^ Because of the probability of rapid disease dissemination, systemic chemotherapy is recommended as the treatment.^3^ Thus, precise and early diagnosis of this unusual neoplasia is fundamental for improving survival rates.

The objective of this study was to report on a case of gastrointestinal multiple lymphomatous polyposis, with a description of the main aspects of the clinical presentation, diagnosis, evolution and treatment of this unusual neoplasia.

## CASE REPORT

**Figure 1:**
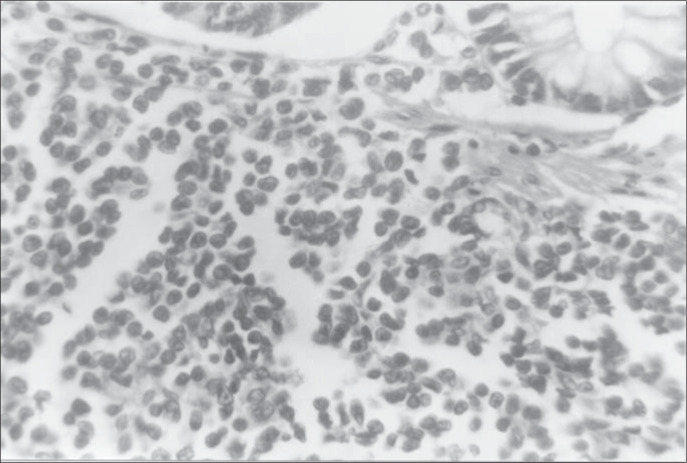
Mantle cell lymphoma in a 59-year-old male patient. Photomicrograph showing diffuse proliferation of small atypical lymphoid cells composed of small cleaved cells with irregular shaped nuclei (hematoxylin and eosin, original magnification x 400).

**Figure 2:**
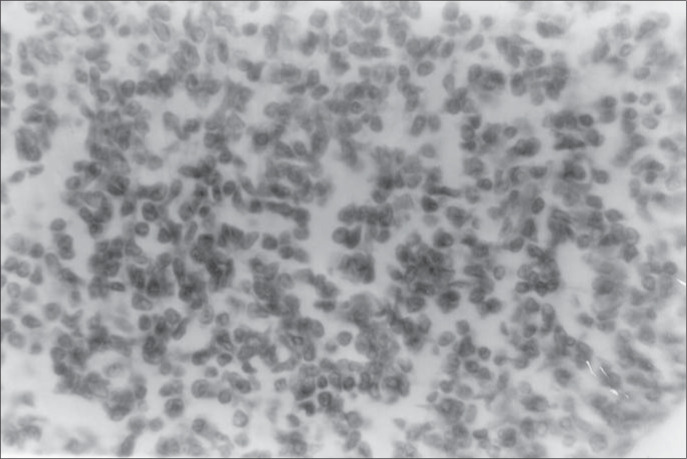
Mantle cell lymphoma in a 59-year-old male patient. Photomicrograph showing neoplastic cells immunostaining for kappa (k) light chain. The lymphoma cells have diffuse positive staining (immunoperoxidase with hematoxylin counterstain, original magnification x 400).

The patient was a 59-year-old white male with a complaint of asthenia, night sweating, alteration in intestinal habit and weight loss of 14 kilograms over the preceding two months. The physical examination showed pallid mucosa and a palpable mass in the epigastrium and mesogastrium. No superficial lymph nodes were palpated. The laboratory tests were normal. Endoscopy of the upper digestive tract showed the presence of gastric and duodenal polyps of length 0.1 to 0.5 mm along their major axis. Anatomopathological examination of these lesions revealed atypical severe lymphocytic infiltrate in the corium. A computed tomography (CT) scan of the abdomen revealed retroperitoneal lymph nodes of increased size and thickening of the gastric wall, while the liver and spleen were normal. An opaque enema showed multiple polypoid lesions, especially in the cecum. Rectosigmoidoscopy demonstrated thickening of the mucous creases, especially in the rectum. A rectal biopsy revealed infiltration of the mucosa and submucosa by diffuse lymphoma consisting of small cleaved cells (Figure 1). Immunohistochemical study of the rectal biopsy showed lymphocytes that expressed the antibody CD20 (L-26), a lymphocyte B marker. The neoplasia cells also expressed light-chain kappa (k) immunoglobulin, but not light-chain lambda (l) immunoglobulin, thus characterizing monoclonal neoplasia (Figure 2). The myelogram was compatible with myeloproliferative syndrome.

The patient presented a condition of acute intestinal obstruction and was submitted to exploratory laparotomy. In the abdominal cavity there was a mesenteric mass formed by agglutinated lymph nodes of increased size that surrounded the proximal ileum, thereby obstructing its lumen. The operation proceeded with the removal of the mesenteric lesion and a segment of the proximal ileum by means of enterectomy and end-to-end enteric anastomosis. Gastrotomy was performed and excisional biopsies were taken from the gastric polypoid lesions. The postoperative period did not reveal abnormalities. Histological examination of the gastric polypoid lesions, mesenteric mass and the segment of small intestine showed the presence of diffuse lymphoma of small cleaved cells (Working Formulation Group classification) that, in the small intestine, were infiltrating the submucosa and muscle tunica.

Around one month after the operation, the patient began chemotherapy, undergoing cycles of the COP+B scheme every 21 days (vincristine 1.4 mg/m^2^ iv, bleomycin 3 mg iv, cyclophosphamide 20 mg iv and dexamethasone 4 mg, all intravenous). During the following two months, the patient progressed in a satisfactory manner, with partial regression of the abdominal masses. After this period, there was a worsening of the general state, with an increase in the dimensions of the abdominal masses and sepsis, accompanied by progressive respiratory insufficiency, leading to death.

## DISCUSSION

Gastrointestinal multiple lymphomatous polyposis is a rare type of gastrointestinal lymphoma with aggressive biological behavior and early systemic dissemination. It most frequently compromises the large intestine, followed by the small intestine, stomach and duodenum. The patient in this report had his stomach, duodenum, small and large intestines compromised simultaneously. The disease occurs more often in men than in women, and it usually appears during the fifth and sixth decades of life.^3,4^

The gastrointestinal lesions of gastrointestinal multiple lymphomatous polyposis are represented by multiple sessile or pedunculate polyps, of a few millimeters to several centimeters in size.^2,3,4^ There is often a dominant ileocecal mass and regional lymphadenopathy,^3,4^ as in the patient we operated on. There may also be involvement of peripheral lymph nodes, bone marrow and peripheral blood.

Gastrointestinal multiple lymphomatous polyposis is histologically characterized by a polypoid accumulation of lymphoid tissue, especially in the submucosa. The polypoid lesions are formed by blocks of uniform lymphoid cells with few mitoses and little phagocytic activity.^1,2^ Characteristically, these lesions do not present germinative centers or follicles. The cell proliferation is limited to the mucosa and submucosa, without destructive invasion of the subjacent tissue. The epithelial coating of the intestinal mucosa remains intact, although it may be a focus for erosion and consequent bleeding.^3,4^ All immunologically investigated cases of gastrointestinal multiple lymphomatous polyposis have been of cell type B.^4^

According to Kiel's classification, gastrointestinal multiple lymphomatous polyposis is classified as a centrocytic lymphoma of B-cells and, according to the Working Formulation Group classification, it is a diffuse malignant lymphoma of small cleaved cells.^2^ Because the prognosis is poor, gastrointestinal multiple lymphomatous polyposis should be considered to be a high-grade lymphoma.

Approximately 90% of the patients with gastrointestinal multiple lymphomatous polyposis present gastrointestinal manifestations that are associated with weight loss, asthenia, fatigue, lethargy, anemia, palpable abdominal or rectal masses and superficial lymphadenopathy. Compromised bone marrow is found in the advanced stage of the disease.^1,2^

The differential diagnosis of gastrointestinal multiple lymphomatous polyposis is made in relation to adenomatous polyps, familial hereditary polyposis, Peutz-Jeghers syndrome, colorectal carcinoma, atypical adenoma and lymphoid nodular hyperplasia with hypogammaglobulinemia. The clinical characteristics, family history and histological characteristics peculiar to each of these diseases distinguish them from gastrointestinal multiple lymphomatous polyposis.^1,2^

When the diagnosis of gastrointestinal multiple lymphomatous polyposis is confirmed, surgical exploration of the abdominal cavity is only indicated in the presence of complications: acute intestinal obstruction, as in the present case, bleeding in the digestive system and peritonitis resulting from intestinal perforation.

The treatment that offers the best results is systemic chemotherapy. If promptly initiated, this may impede the characteristic early systemic dissemination of the disease and reduce the size of the tumors.^1,2,4^ The cyclophosphamide, doxorubicin, vincristine and prednisone (CHOP) and cyclophosphamide, vincristine and prednisone (COP) schemes are the ones most often utilized.^2^ According to Ruskoné-Fourmestraux et al.,^4^ a strategy combining a standard anthracycline-containing multidrug regimen and intensification with total body irradiation, supported by autologous stem cell transplantation, appears to be a valid approach for younger patients.

The prognosis for gastrointestinal multiple lymphomatous polyposis is poor, even when the affection is correctly diagnosed and the patient is submitted to chemotherapy. After the diagnosis, the average survival of such patients is usually less than three years.^1,3,4^ Even so, accurate diagnosis of gastrointestinal multiple lymphomatous polyposis and immediate chemotherapy are important measures in attempting to impede early systemic dissemination of the disease and diminish the high mortality rate among patients with this unusual neoplasia.
